# A systematic literature review on the use of big data analytics in humanitarian and disaster operations

**DOI:** 10.1007/s10479-022-04904-z

**Published:** 2022-11-21

**Authors:** Abhilash Kondraganti, Gopalakrishnan Narayanamurthy, Hossein Sharifi

**Affiliations:** University of Liverpool Management School, Chatham Street, Liverpool, L69 7ZH UK

**Keywords:** Humanitarian, Disaster, Big data, Analytics, Systematic literature review

## Abstract

At the start of this review, 168 million individuals required humanitarian assistance, at the conclusion of the research, the number had risen to 235 million. Humanitarian aid is critical not just for dealing with a pandemic that occurs once every century, but more for assisting amid civil conflicts, surging natural disasters, as well as other kinds of emergencies. Technology's dependability to support humanitarian and disaster operations has never been more pertinent and significant than it is right now. The ever-increasing volume of data, as well as innovations in the field of data analytics, present an incentive for the humanitarian sector. Given that the interaction between big data and humanitarian and disaster operations is crucial in the coming days, this systematic literature review offers a comprehensive overview of big data analytics in a humanitarian and disaster setting. In addition to presenting the descriptive aspects of the literature reviewed, the results explain review of existent reviews, the current state of research by disaster categories, disaster phases, disaster locations, and the big data sources used. A framework is also created to understand why researchers employ various big data sources in different crisis situations. The study, in particular, uncovered a considerable research disparity in the disaster group, disaster phase, and disaster regions, emphasising how the focus is on reactionary interventions rather than preventative approaches. These measures will merely compound the crisis, and so is the reality in many COVID-19-affected countries. Implications for practice and policy-making are also discussed.

## Introduction

Humanitarian crises have been on the rise (UN OCHA, 2020), and, due to the increasing complexity of human societies, are threatening societies' livelihood more than ever. According to UNDRR (2020b), the number of natural disasters has doubled from the period of 1980–1999 to the period of 2000–2019. Response to the events and crises costs the global society some extensive amounts (e.g., according to Financial Tracking Service (2021), the funding requirements in 2020 were estimated at $38.54 billion). While accessing such funds is increasingly challenging to provide for, the bigger issue is the cost-effectiveness in operations to prevent excessive reliance solely on funding.

Generally, as most aspects of the modern society, use of new and emerging technologies has been a major part of the new solutions to old and new problems. For instance, disaster relief operations are mainly logistical, accounting for 60 to 80% of total humanitarian relief spending (Lacourt & Radosta, 2019; Van Wassenhove, 2006). Owing to the lack of analysis and relief efforts duplication, it is estimated about 35 to 40% of these logistical expenses are frittered (Day et al., 2012; Kwapong Baffoe & Luo, 2020). The crucial, uncertain, and intricate nature of field operations necessitates swift decision-making (Knox Clarke & Campbell, 2020). Furthermore, the field of humanitarian and disaster operations (HDO) is diversifying with the engagement of individuals including volunteers and crowdsourcing participants who may not be closely associated or affiliated with humanitarian organisations and lack adequate training. As a result, the deployment of new technologies, particularly big data analytics (BDA), has become a critical component in resolving concerns about collaboration, efficiency, and efficacy in crisis and relief operations (Dubey et al., 2019; Jeble et al., 2019; UN OCHA, 2021). HDO has seen considerable transformations over the years, from traditional volunteers to digital volunteers (Behl et al., 2021a, 2021b), and from conventional donations to technology-driven crowdfunding platforms (Behl & Dutta, 2020; Behl et al., 2021a, 2021b).

Evidently, the application of BDA in the humanitarian and disaster sector has been rare (Centre for Humanitarian Data, 2019), and way behind the commercial business sector. On the other hand, usefulness of these technologies has been a matter of debate. As examples, while Swaminathan (2018) argues that, incorporating BDA may also enable humanitarian organisations in experiencing operational improvements, Sharma and Joshi (2019) opined that data does not accurately reflect the situation on the ground, and relying significantly on BDA may undermine humanitarian operations. A line of disagreement can be the social and human sides of humanitarian operations, where the core humanitarian principle of being humane (UN OCHA, 2010) might be challenging to achieve if sent in a data-driven non-human context.

In our study, HDO are defined as operational activities involved in any stage of a humanitarian crisis or disaster, including mitigation initiatives, preparedness efforts, relief-related activities, and recovery associated actions. The future of HDO in the light of BDA is an important topic, which has been partially addressed with many questions and challenges to engage with and answer. While the literature on the subject has been growing, it still does not encompass all of the existing collective views, challenges, aspects of the use of new technologies (BDA here), and ways ahead for the sector. Such challenges are further intensified when considering the scope and depth of the problems in hand including: types of disasters; contextual aspects of the problem such as geographical, social and economic issues; and complexities of the process to adopt, successfully apply and manage implications of the new technologies.

The academic research domain is yet to become mature on the use of BDA in the HDO field. The use of technology in HDO witnessed a surge at some point, particularly after the 2010 Haiti earthquake (Burns, 2015; Ragini et al., 2018; Read et al., 2016; Sandvik et al., 2014), but still remains as a discussion point largely. After a decade, another disaster, COVID-19, as an unprecedented event, has brought the attention back on BDA where data driven decision making is significantly increased (Gazi & Gazis, 2020). But what has happened in the last 10 years, how far have we come in this field, and what key issues are there for the research community to consider that need new insight and answers. This research attends this matter and attempts to review the state of academic research on BDA in HDO. The article aims at delivering a thorough review of the subject matter as well as insights into areas where the future research could focus. The main objective of this review is to examine and evaluate how BDA has been employed in numerous disasters, disaster phases, and disaster locations in the field of HDO to assess how far the research has progressed so far. To achieve this, three research questions (RQ) are designed for this study as follows:

- **RQ1.** How has the research on the application of BDA for HDO evolved over time?
- **RQ2.** What is the status of the BDA application across different disaster categories, disaster phases, disaster locations, and what different types of big data have been used?
- **RQ3.** What are the key theoretical lenses used to examine and explain BDA application in HDO?

The study contributes to the subject domain by offering a research background to understand the state of disasters and the review of extant literature reviews in the field. The method utilised to undertake the review, including the review protocol, search strategy, and article quality assessment, is outlined in the following section. The outcomes of the review are reported and discussed in the sections that follow. Finally, the paper offers areas for further research to enhance the application of BDA in the HDO sector, as well as the review's limitations.

## Research background

This research integrates humanitarian crisis and disaster operations together. Humanitarian operations takes place to alleviate human suffering where local mechanisms are inadequate to accommodate and offer the necessary assistance (ReliefWeb, 2008). Disaster operations, on the other hand, include activities carried out before, during, and after a disaster to save lives, reduce economic damage, and restore normalcy (Altay & Green III, 2006). This section discusses the current state of different disaster categories, and evaluates existing literature reviews in the field to assess the field's progress.

### State of the disaster types

Before getting into the actual review, this study needs to understand what types of disasters are out there and how these are classified over the years in order to report disasters in review articles in the form of a standardised list. Besides, adhering to the standard list of disasters leads to better reporting and ease in comparisons.

The exploration revealed that there is no particular norm when it comes to disaster types. Scholars initially described disasters into two types, ‘natural’ and ‘man-made’ (Berren et al., 1980; de Boer, 1990) or ‘natural’ and ‘human-induced’ (Gray, 1982) but the new type of disasters ‘industrial’ (Taylor, 1990) and ‘hybrid’ (Shaluf et al., 2001; Shaluf, 2007a, 2007b) are added to the list at later years. Altay and Green III (2006) in their review of disasters in operation management separated disasters mainly into natural and man-made and the continuation review by Galindo and Batta (2013) also retains the same description for disasters. These are again altered in the last decade and changed the description to natural and human-made or human-induced disasters (Khan et al., 2020). Disasters in the twenty-first century are never constant as the human race has witnessed and is continuously witnessing new and different kinds of modern disasters in this century (De Smet et al., 2012). Hence the type of disasters is changing over the years. Eshghi and Larson (2008) reviewed twentieth-century disasters to build a new classification and described that the variance in initial classifications is due to the difference in describing the disasters and their impacts. Although the categorisation is inconsistent and changing over time, natural disasters and human-induced disaster categories are commonly used and considered as a broader generic group.

As Lukić et al. (2013) suggested, natural disasters can be categorised based on the physical cause of the incident. Further, a common classification is necessary to have global standards and this will help in assessing disasters without any hazard bias, threshold bias, and accounting bias. Guha-Sapir and Below (2002) assessed and compared three well-known global disaster datasets EM-Dat (by CRED), NatCatSERVICE (by Munich Re), and Sigma (by Swiss Re). One of the key issues that surfaced from this comparison is the lack of standardisation of methods and definitions. These differences were mainly attributed to the discrepancies in disaster typology. To overcome this, disaster databases EM-Dat and NatCatSERVICE have come together to implement a standard disaster classification which is reviewed and agreed upon by other databases and OCHA (Wirtz et al., 2014). The new classification provides two generic categories of natural and technological, which comprise the entire disaster spectrum. The first generic category, natural disasters are further divided into six groups namely biological, climatological, extraterrestrial, geophysical, hydrological, and meteorological. The second generic category technological disasters, is in the place of human-induced disasters and covers three groups; industrial, transport, and miscellaneous (Guha-Sapir, 2008). The new classification hierarchy is established on a ‘triggering event’ logic (Below et al., 2009). The same classification is implemented for CRED’s annual disaster statistical review from 2007 reports and followed by many other databases.

**Fig. 1:**
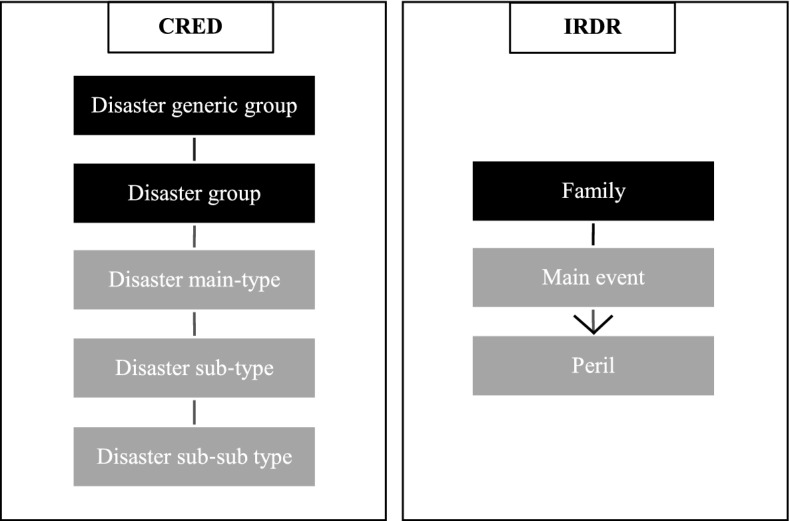
Disaster classification: CRED (2008) by Guha-Sapir (2008) versus IRDR (2014). *Source*: compilation by author

However, Integrated Research on Disaster Risk (IRDR) program sponsored by the United Nations Office for Disaster Risk Reduction (UNDRR) tested the operating viability of the new classification provided by CRED and Munich Re in national databases, and concluded that implementation of this classification in national databases is difficult. The reason given was that national databases run primarily at the peril level and CRED classification is more of a top-down approach where bottom-level disaster types are exclusively associated with sub-types therefore to main types, as shown in Fig. 1. This allowed IRDR to work on revising the existing framework. The relationship between peril and main disaster event is not exclusive in the revised classification meaning perils can be linked to multiple disaster categories in the main event as illustrated in Fig. 1. However, the main level classification of natural disasters remains the same in the IRDR classification (IRDR, 2014).

The bottom-level classification is such an uneven segment in the disaster typology, it changes from time to time from one event to another depending on the definite occurrence and causes for loss. A great deal of work has gone into the CRED's disaster classification since the beginning of the twenty-first century, and their initiation through EM-Dat to improve and standardise the classification has opened doors for academicians and United Nations (UN) organisations to try and implement the disaster classification in their area of work.

**Fig. 2:**
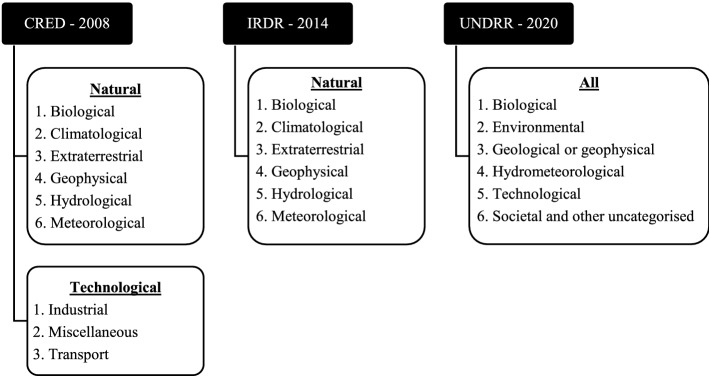
Comparison of the first-level classification between CRED versus IRDR versus UNDRR. *Source*: compilation by author

IRDR is only focused on natural events and the UNDRR’s latest work is dedicated to all event approach following the Sendai Framework (UNDRR, 2020a). The new list has avoided a hierarchical approach in classifying disasters, citing the dynamic relationship between various events will be inadequate in hierarchical style and preferred non-hierarchical or flat list (UNDRR, 2020a). Figure 2 depicts the generic and group-level disasters in CRED, IRDR, and UNDRR.

Global disaster databases and UNDRR classified disasters based on the causative dimension and this has been the popular choice. This study is not looking into the peril level classification for the categorisation of disasters in articles but only takes into consideration of the disaster generic group (e.g. natural disaster) and the first level disaster group (e.g. geophysical). This review will be using CRED’s classification of natural disasters as it is simple, distinguishes between all-natural disasters, and more importantly separates from non-natural disasters. The remaining disasters in the review will be identified as human-induced disasters.

### Review of reviews

There have been no reviews in the field of BDA and HDO before 2016. Although the research in the field has been marginal over the years, it has recently accelerated as a result of the volatile world we now live in. Furthermore, this discipline is becoming more interconnected and multidisciplinary, making it difficult to keep up with the ongoing research and remain on the cutting edge (Snyder, 2019). This research has revealed 13 review studies and surveys of the literature conducted thus far, of which an examination shows that 77% of these studies are not comprehensive. This means the studies either only look at one type of disaster (Balti et al., 2020), one particular disaster phase (Cumbane & Gidófalvi, 2019), one form of big data source (Wang & Ye, 2018), one element of disaster (Sarker et al., 2020a), or the combination of multiple technologies (Khan et al., 2020). Table 1 summarises all thirteen studies identified and briefly describes each review's emphasis. The identified reviews are of several forms, including systematic literature review (SLR), literature review (LR), literature survey (LS), and systematic literature survey (SLS).

**Table 1:** Total number of existing reviews in the field. *Source*: compilation by author

Review article	Author (year)	Review focus	Review type
Big data analytics for emergency communication networks: A survey	Wang et al. (2016)	Survey on the combination of technologies	SLS
Social media analytics for natural disaster management	Wang and Ye (2018)	Review on one type of big data source, and one type of disaster generic group	LR
A review on application of data mining techniques to combat natural disasters	Goswami et al. (2018)	Survey on one type of disaster generic group	LS
Big data and disaster management: a systematic review and agenda for future research	Akter and Wamba (2019)	Full-scale review	SLR
Big data in humanitarian supply chain management: a review and further research directions	Gupta et al. (2019)	Full-scale review	SLR
The rising role of big data analytics and IoT in disaster management: Recent advances, taxonomy and prospects	Shah et al. (2019)	Review on the combination of technologies	SLR/LS
Review of big data and processing frameworks for disaster response applications	Cumbane and Gidófalvi (2019)	Review on one type of disaster phase	SLR
Challenges of using big data for humanitarian relief: lessons from the literature	Sharma and Joshi (2019)	Full-scale review	LR
Climate change adaptation and resilience through big data	Sarker et al. (2020b)	Review on one type of disaster group	SLR
Multi-hazard disaster studies: Monitoring, detection, recovery, and management, based on emerging technologies and optimal techniques	Khan et al. (2020)	Survey on multiple technologies	SLS
Big Data and Emergency Management: Concepts, Methodologies, and Applications	Song et al. (2020)	Survey on multiple technologies	LS
Disaster resilience through big data: Way to environmental sustainability	Sarker et al. (2020a)	Review on one element of disaster management	SLR
A review of drought monitoring with big data: Issues, methods, challenges and research directions	Balti et al. (2020)	Review on one type of disaster	LR

A review study on the intersection of BDA and HDO is considered a full-scale review. Full-scale literature review papers are very few in this field, and only three papers, Akter and Wamba (2019); R1, Gupta et al. (2019); R2, and Sharma and Joshi (2019); R3 have been identified from the list. The first two reviews, which were conducted around the same time, offered more positive perspectives by outlining the benefits and emphasised the need for use of big data in a humanitarian and disaster setting. The third review has attempted to bring the arguments of the challenges and negative effects related to the use of big data in relief operations. The search string that was used to shortlist the studies is clearly stated in R1 and R2, but this was not the case in R3, which stated that studies were obtained using several databases, but did not include the search string that could help scholars reproduce the results for further verification. The period was flexible in these review papers, R2 and R3 did not restrict themselves to a specific period, however, R1 acknowledges that it did come across very few articles before 2010, therefore it chose to focus on studies published between 2010 and 2017. The primary reason for undertaking an SLR on top of the already existing three papers is that close to 70% of articles on the review topic are published in the last 3 years, meaning, after the research conducted for R1 and R2. More specifically, our study contains only seven articles that are reviewed in either R1 or R2. This number further approves the necessity for an SLR in the field to revisit the review areas that were not covered in R3 (even if they were covered in R1 and R2) such as Classification: by research methodologies, Classification: by disaster phase, Disaster occurrence (year), and Theoretical underpinnings. The blind eye on the management subject area is evident in which, R1 papers from the management field are just above five and in R2 the number is below five. There is a marginally better number in the current study with roughly 10% of papers coming from the management domain but it is nowhere near the top two preferred subject areas of the field.

Besides a few similarities and a good range of dissimilarities in the inclusion and exclusion criteria between the first two review papers, the theoretical underpinning debate is discussed in both studies. R1 explicated the lack of representation of theories in the field and offered some ideas on a few theories as a future research direction. On the other hand, understanding the field from the organisational theoretical lens is one of the research objectives of R2. This study can’t stress enough the importance of theoretical requirements in the field of BDA in HDO. Although R3 was not forthcoming in presenting the important aspect of search criteria that is required for any review, it does stand as the inimitable review in this nascent field as it brings a different view of big data in humanitarian relief, called negative effects. The review divided the articles into three groups: supportive, mixed, and critical. Drawing upon the critical section, a total of eight challenges were discussed. Some challenges are related to ethical concerns, errors caused by either language or culture, and issues with the existence of big data itself.

The three full-scale reviews along with the current review are compared in Table 2 to see how the full-scale reviews are advancing in the field of BDA and HDO. The assessment is based on the review results, and how the authors classified the extant research in their review. The review area named as ‘distribution’ in the table is descriptive where the distribution of articles is available from the respective database they chose for review. Because descriptive results were not considered for this review, we will not present any distribution categories. The remaining review areas which are highlighted in bold are the compilation of outcomes that emerged after reviewing the set of papers. Being the very first full-scale review in the field, R1 mostly produced a basic analysis while at the same time classified papers in three different review areas. R2 which is focused on the humanitarian supply chain papers provided less descriptive outcomes and more analysis on the review papers with supplying enablers and concerns for big data. R3, which was published after the first two reviews, emerged as the less descriptive one and classified articles with real case disasters and reference to the data sources used in the papers. This review will provide a comprehensive overview of the field, incorporating the lessons learnt from the previous three reviews. The reader should bear in mind that this table does not in any way measure the quality of these reviews.

This study identified seven review areas to examine, and these areas were chosen logically to represent the two review themes, HDO and BDA. To begin, it is essential to analyse the event in terms of what it is (disaster type), what stage it is in (disaster phase), and where it occurred (disaster location) from the aspect of disaster/humanitarian crisis management. We added 'when it occurred' (disaster year) to this to observe how scholars choose events; recent or historical disasters. Then, from the standpoint of BDA, we are interested in the types of big data (sources of big data) that have been used/examined in previous studies. We still regard this as a nascent field, thus we provided the types of research (research methodologies) undertaken in the field as well as the theories (theoretical underpinnings) that are applied to assess how far we have come.

**Table 2:** Comparison between three reviews in the field. *Source*: compilation by author

Review area	R1	R2	R3	This review
Distribution: by authors	✓	✘	✘	✘
Distribution: by universities	✓	✘	✘	✘
Distribution: by countries	✓	✘	✓	✘
Distribution: by subject areas	✓	✓	✘	✘
**Classification: by research methodologies**	**✓**	**✓**	**✘**	**✓**
**Classification: by disaster phase**	**✓**	**✘**	**✘**	**✓**
**Classification: by research (data) cluster**	**✓**	**✘**	**✘**	**✘**
Distribution: source title	✘	✓	✘	✘
**Big data enablers**	**✘**	**✓**	**✘**	**✘**
**Big data concerns**	**✘**	**✓**	**✘**	**✘**
**Classification: by disasters or disaster categories**	**✘**	**✘**	**✓**	**✓**
**Classification: by source of big data**	**✘**	**✘**	**✓**	**✓**
**Classification: by argument (supportive, mixed, critical)**	**✘**	**✘**	**✓**	**✘**
**Disaster locations**	**✘**	**✘**	**✓**	**✓**
**Disaster occurrence (year)**	**✘**	**✘**	**✘**	**✓**
**Theoretical underpinnings**	**✓**	**✓**	**✘**	**✓**

## Methodology

A systematic literature review (SLR) is a research approach that is used to gather and critically evaluate the current state of knowledge on the study topic to address research questions. SLR was implemented as a result of four important considerations. First and foremost, it seeks to provide clarity to the overall process through the use of a review protocol and a carefully planned search strategy (Booth et al., 2012). Second, the authors wish to prevent any bias in performing the study, particularly selection and publication bias, and SLR principles can help to reduce this and facilitate the development of more accurate results (Becheikh et al., 2006). Third, it must be transparent throughout the review process (Booth et al., 2012) and, fourth, it has to be reproducible for other researchers interested in extending this research (Booth et al., 2012). The principles of Denyer and Tranfield (2009) and Tranfield et al. (2003), two commonly employed SLR techniques in management, were adopted in this review, and they are also preferred in the operation and supply chain domain (El Baz et al., 2019; Gligor & Holcomb, 2012; Tachizawa & Wong, 2014).

**Fig. 3:**
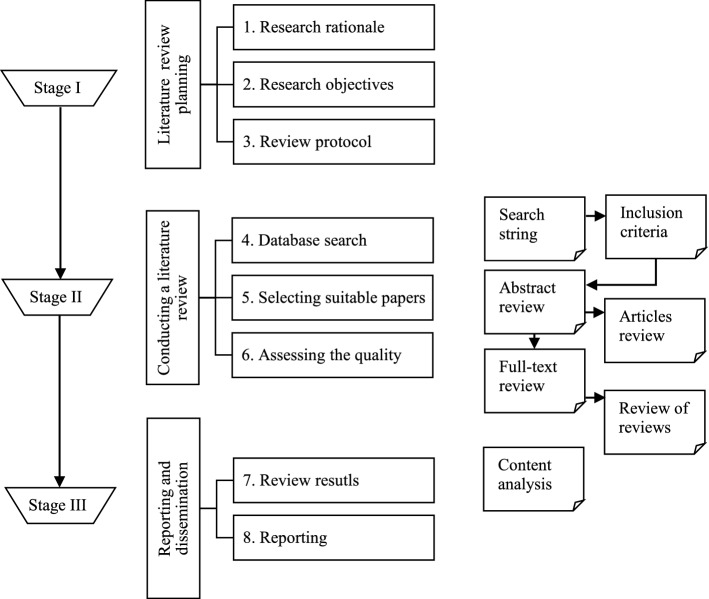
Systematic literature review process. *Source*: Adopted from Tranfield et al. (2003) and Denyer and Tranfield (2009)

### Review protocol

The research protocol facilitates the execution of the second stage of the study, 'conducting a literature review,' which is the fundamental component of this research in the SLR process depicted in Fig. 3. The goal of this protocol is to eliminate any researcher bias (Tranfield et al., 2003), therefore a search strategy with a clear set of rules is in place to find the relevant journal articles for this study. As a result, the search for existing literature is facilitated by the selection of a more appropriate citation database, and Scopus was selected for this review. Scopus is regarded as the most comprehensive multidisciplinary database, with more journal coverage than Web of Science (Aghaei Chadegani et al., 2013).

### Search strategy

The search strategy used to shortlist academic literature utilising inclusion and exclusion criteria determines the efficacy of SLR (Snyder, 2019). Using the Boolean operators, a search string in Scopus was created which represents both BDA and HDO in the search results. The authors are cautious that inserting more keywords may significantly narrow the search and perhaps omit any relevant literature. As a result, the search string is not rigorous and is as broad as feasible. Because this is a rapidly evolving field, and as a measure, authors are mindful in selecting search keywords. BDA is split into two terms: 'big data' and 'analytics,' because some research papers might have used either name in the keywords, abstract, or title rather than the complete phrase BDA. These keywords are linked with two others, "humanitarian" and "disaster," which represent the field of HDO. The complete search string that was used is listed below.

(("analytics" AND "humanitarian") OR ("analytics" AND "disaster") OR ("big data" AND "humanitarian") OR ("big data" AND "disaster"))

**Fig. 4:**
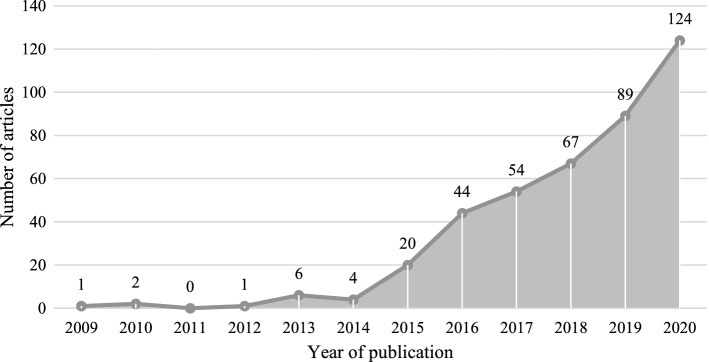
Research publication over the years by the number of articles. *Source*: compilation by author

The search criteria, as indicated in Table 3, consist of five levels that have aided in the selection of relevant articles, and this was executed within Scopus. The search string was used in the search area, which resulted in 1,563 articles in the first level. Due to the multidisciplinary nature of the study field, five suitable subject areas have been included at level two, bringing the total to 1,354. Only peer-reviewed articles are considered in this review, limiting the total to 483 at level three. The rationale for analysing solely published material is that it can improve the review's quality because most publications undergo a thorough peer-review process (Light & Pillemer, 1984). Additionally, the number dropped to 468 when only journal papers are considered at level four. Finally, filtering our search to papers written in English yields a total of 417 articles. Although there was no constraint on publication year during the search, the earliest paper can be tracked back to 2009, as seen in Fig. 4. The data collection procedure began in April 2020, with the first search conducted on April 29th, and the follow-up searches conducted on July 23rd and December 31st of the same year to update the sample.

**Table 3:** Search criteria results in Scopus. *Source*: compilation by author

Level	Criteria	Description	Results
L1	Search area	Title, keyword, abstract	n = 1563
L2	Subject area	Computer science, engineering, decision sciences, social sciences, business, management and accounting	n = 1354
L3	Document type	Article, review	n = 483
L4	Source type	Journal	n = 468
L5	Language	English	n = 417

## Abstract and full-text review

An additional shortlisting process is used by evaluating the search results employing inclusion and exclusion criteria. The abstracts of 417 papers were thoroughly studied, but, when authors thought that the abstract content was insufficient to establish the article's relevance, a full-text review was undertaken. This procedure eliminated around 62% of the papers, leaving 160 for full-text review. One example of an article that has been omitted is ‘Predicting Heart Diseases from Large Scale IoT Data Using a Map-Reduce Paradigm’ (Abd & Manaa, 2020). While this article does not discuss humanitarian or disaster operations, it was surfaced in the list due to the inclusion of the key terms ‘big data’ and ‘disaster’ in the abstract.

This study is concept-centric, with a framework designed to capture the key themes in each study to achieve comprehensiveness (Webster & Watson, 2002). For full-text papers, the inclusion criterion is based entirely on one parameter; ‘Is the article at the intersection of BDA and HDO?’ This evaluation has been carried out by classifying articles into three distinct categories. Table 4 shows that category one has the most relevant publications to the study topic. For instance, Dubey et al.’s (2018) article titled ‘Big data and predictive analytics in humanitarian supply chains: Enabling visibility and coordination in the presence of swift trust’ focused on both humanitarian and BDA, hence listed in category one. Category two, on the other hand, is marginally relevant and one such example for this category is ‘Disaster management in the digital age’ (Talley, 2020), which discusses various technologies that can be used in disaster management, including BDA. Wherein articles from category three are unrelated and do not contribute to the advancement of this review. If we look at Mann’s (2018) paper, ‘Left to Other Peoples’ Devices? A Political Economy Perspective on the Big Data Revolution in Development’, it shifts data 4 development (D4D) focus to the economic development, hence placed in category 3. This review considered articles from categories 1 and 2, containing 86 studies, 13 of which were reviews. We opted to produce findings for conceptual, empirical, and model papers, totalling 73 articles.

**Table 4:** Full-text review results. *Source*: compilation by author

Category	Description	Results
1	The focus of the article is on HDO and BDA as the key point	n = 54
2	Considerable insights in the article on the intersection of BDA with HDO	n = 32
3	The article is not relevant to the research area	n = 74

## Results

The authors report important findings from the final set of papers in this section, which were identified following fit assessment criteria and are structured into seven review areas in six sub-sections. First category outlines which disasters are more concentrated and where the research is inadequate. The second category reveals which disaster stages are more popular among academics. The third category focuses on disaster locations, as well as how many of these are on real-world disasters and their group. The fourth category is about the big data sources utilised to perform the research and which of these are common in each disaster phase. The fifth category briefly discusses studies associated with theories. At the end of the section, results allied with research methodologies utilised in articles are also presented.

### Disaster categories

**Fig. 5:**
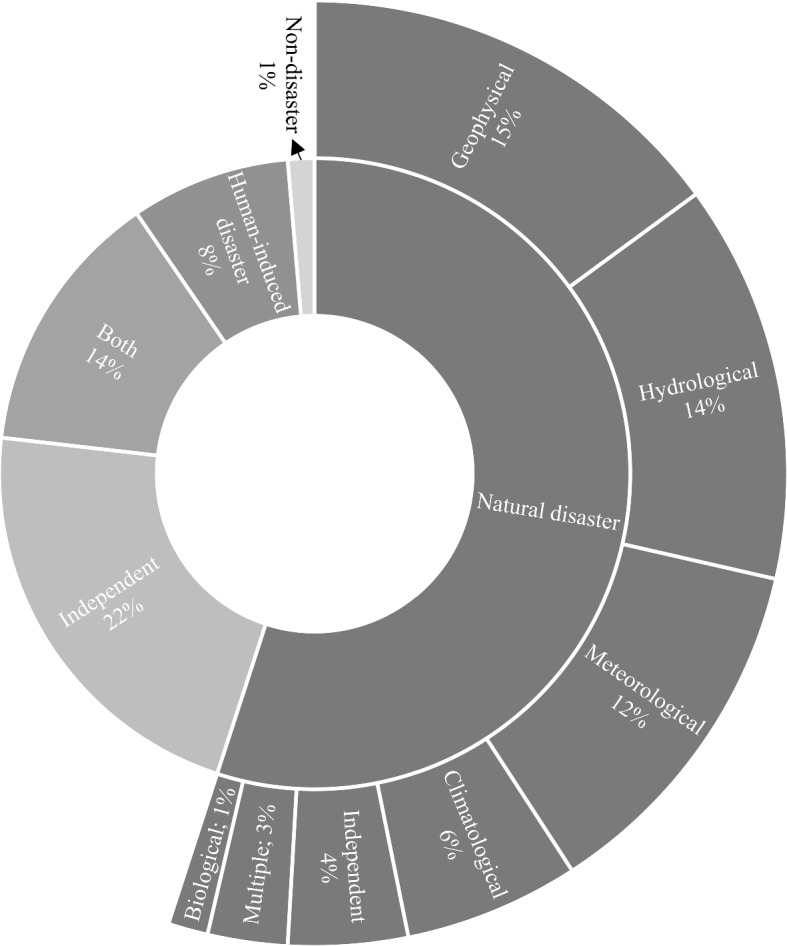
Disaster group in articles-separated by generic and first-level disaster group. *Source*: compilation by author

Scholars had put more importance on natural occurrences, as seen in Fig. 5 because natural disasters comprise more than half of disasters reviewed in the literature. Within the first generic group 'natural disasters', geophysical disasters such as volcanic activity, earthquakes, and tsunamis, along with hydrological disasters including floods and heavy rains were studied. Floods and earthquakes are the predominant choices for researchers in this category of sudden-onset disasters. The interest in geophysical disasters revolves around situational awareness prior to the disaster (Amato et al., 2019), public emotion (Yang et al., 2019), supply chain resilience (Papadopoulos et al., 2017), and information exchange behaviour (Li et al., 2018). Further, demand estimation for shelters (X. Zhang et al., 2020b), and the development of an information system to assist logistic operations in reaching the affected people (Warnier et al., 2020) were prioritised. Scholars investigated various aspects of the hydrological disaster group, including responding to the disaster through sentiment analysis (Ragini et al., 2018), bridging the information gap between responding organisations (van den Homberg et al., 2018), and understanding the severity of the disaster (Kankanamge et al., 2020). In addition, academics were interested in forecasting the disaster (Puttinaovarat & Horkaew, 2019), and estimating the need for relief supplies (Lin et al., 2020) in the hydrological group. Researchers are also paying attention to another sudden-onset disaster group, meteorological disasters which include hurricanes and typhoons. The research in this group focuses on understanding the needs of impacted people and how their priorities change (Malawani et al., 2020), examining the societal impacts (Zhang et al., 2020a), understanding human activities in disasters (Liu et al., 2020), sociodemographic factors influencing disaster response (Fan et al., 2020), and public behaviour (Chae et al., 2014). The research in these three disaster groups is quite diversified, and much emphasis has been placed on them, not only because they are more common, but also because of the economic damage and fatalities that they inflict. The work in these three disaster groups is entirely empirical and model based, with the majority of them (77%) focused on real disaster cases.

The group of climatological disasters has received very little attention, with a focus on wildfire and study on the heatwave. Further biological disaster group research is insignificant with only one publication addressing the epidemic crisis. In their research, a couple of scholars focused on multiple disasters inside the natural disaster generic group, with earthquake being one of the multiple disasters. The remainder of papers under the independent category of natural disasters are generic and not particular to any disaster group. Human-induced disasters have rarely been examined; as per the Swiss Re (2021) report, 37% of disasters reported in 2018 were caused by humans, with a 10-year average of more than 30%. However, researchers' interest in this area is negligible. Bahir and Peled (2016) attempted to identify the location of the conflict in their research by analysing textual messages, whereas Rogstadius et al. (2013) and Zhang et al. (2016) studied situational awareness during civil war and riots, respectively. There is a potential in the human-induced disaster segment and big data such as satellite imagery and mobile data that could be significant for those working in the field and monitoring the trends of the situation to better act. These talking points, however, must be translated into better research and then tested in the field. Further, several articles did not cover either of the disaster generic groups, and this contains conceptual and empirical work mainly related to the general humanitarian supply chain, ethics, and privacy. In one publication, the technology was evaluated in a non-disaster context, therefore it was not allocated to any of the disaster groups. There are also articles on the mix of natural and human-induced disasters in which the majority of them are general and discussed humanitarian principles (Sandvik et al., 2017), and humanitarian data sets (Bell et al., 2021).

### Disaster phase

**Fig. 6:**
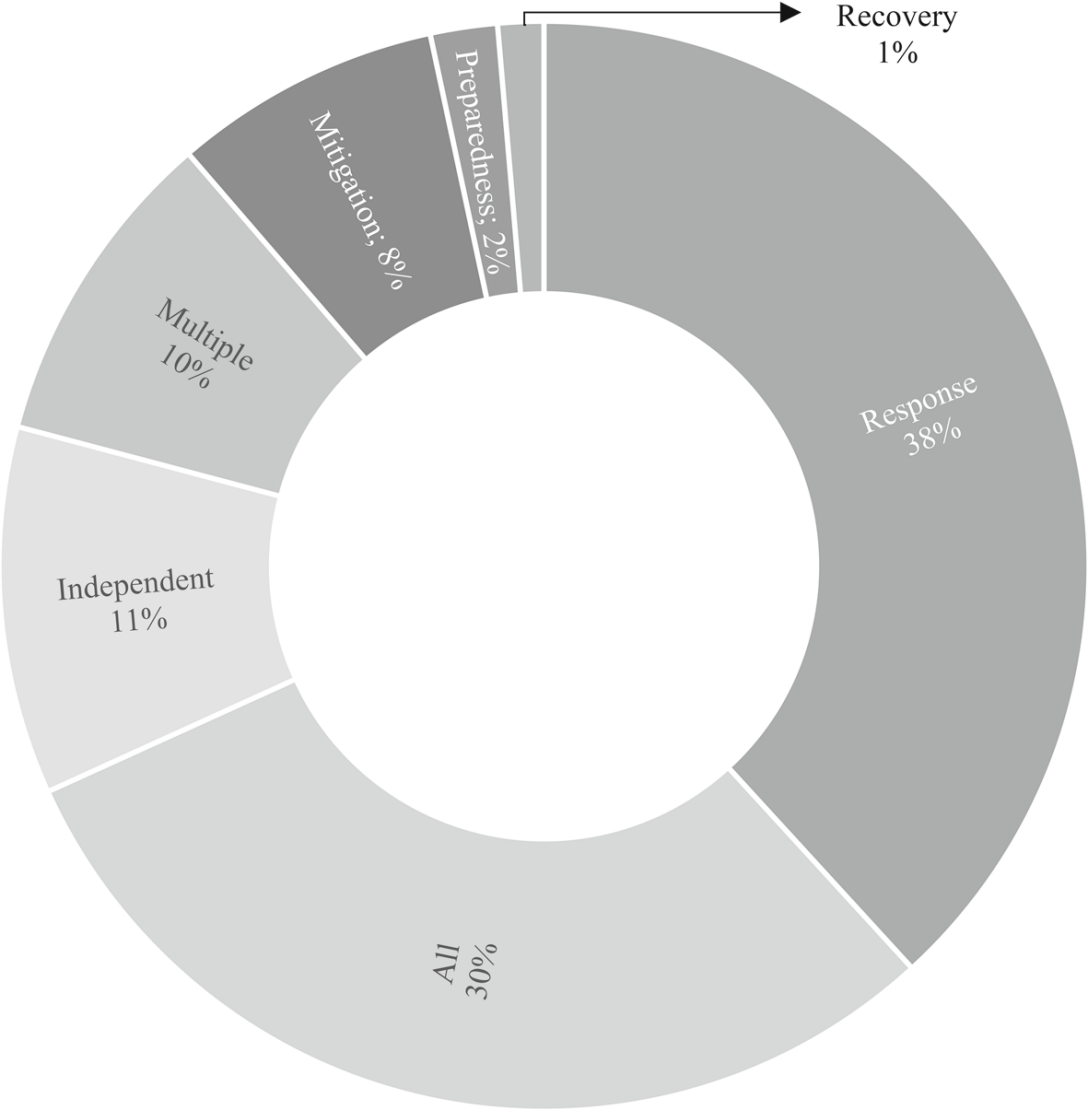
Articles based on disaster phase. *Source*: compilation by author

Disaster occurrences and scenarios in the previous research are divided into four phases-mitigation, preparedness, response, and recovery (Cumbane & Gidófalvi, 2019; Kankanamge et al., 2020; Sarker et al., 2020a). Figure 6 illustrates the articles distribution across these four stages, as well as the inclusion of additional categories, where the combined number of articles from mitigation, preparedness, and recovery is not even a third of the total number of articles from the response stage, thereby demonstrating a drastic imbalance in research between the four stages. The work done thus far in the mitigation phase has primarily focused on two aspects. The first is nowcasting disaster impact and disaster forecasting to mitigate significant risks (Avvenuti et al., 2017; Puttinaovarat & Horkaew, 2019; Qayum et al., 2020), and the second is gaining a better knowledge of people's emotions and situations to assist in minimising the impact (Amato et al., 2019; Yang et al., 2019; Zamarreño-Aramendia et al., 2020). In the preparedness phase, Bag et al. (2021) sought to identify the barriers in employing BDA in the humanitarian supply chain, as well as their interrelationships. This empirical work is timely because there is less research in the preparedness stage in the context of BDA in HDO, and it should help to broaden the conversation. Moreover, research on disaster preparedness in the event of a sudden-onset disaster has to be considerably increased. Because the preparation window is much shorter in this scenario, near-real time and real-time data are more significant. Scholars' top priority over the years has been response events, and this is same for practitioners and policymakers. The response phase is the most intensive, and the established mechanisms will be more overwhelming in this phase than in any other phase. As a result, the disaster response articles in extant research covered all disaster groups except biological, utilised all types of data sources, and spanned across all regions. The focus needs to shift as acting early on can have substantial results on HDO. According to the Boston Consulting Group (2015) report, financial benefits can be as much as double, which implies that spending one dollar before a disaster can save two dollars during the response, and it can also save 1 week of response time on average. This anticipated action may also result in saving lives. Articles focused on more than one phase categorised as multiple and they used the same source of big data, social media (SM). Though this segment is the combination of multiple phases, they all are centred on the combination of response-recovery (crisis management), with only one research focusing on preparedness-response-recovery. The authors' focus during these crisis management phases is on evaluating the sentiments of the affected people, the severity of disaster damage, and data management procedures. The work of Shan et al. (2019) is stimulating in that their model for measuring disaster damage evaluated both physical and emotional damage to people in real-time.

A significant amount of research talk about the complete cycle of disaster with nearly half of them being conceptual papers. Of these, the majority of work is generalised dialogue and articles focused on organisational mindfulness (Amaye et al., 2016) and group privacy (Gerdes, 2020). Also, it is worth noting the work of Iglesias et al. (2020) on building reference architecture for big data, as well as critical components required for the system. Furthermore, the authors highlighted the potential uses of big data in each crisis phase for a variety of tasks based on the core capabilities developed by the National Response Framework. The empirical and model work, on the other hand, concentrated on significant conditions across phases such as coordination in supply chain (Dubey et al., 2018, 2019), crisis communication (Jin & Spence, 2020; Kibanov et al., 2017), and understanding public behaviour (Chae et al., 2014). However, a considerable number of publications did not examine any disaster phase(s), hence classified into the independent category. This category includes papers on the hype around big data (Read et al., 2016), challenges (Bell et al., 2021), big data in digital humanitarian practices (Burns, 2015), and ethics of big data (Taylor, 2016).

### Disaster locations and occurrence

**Fig. 7:**
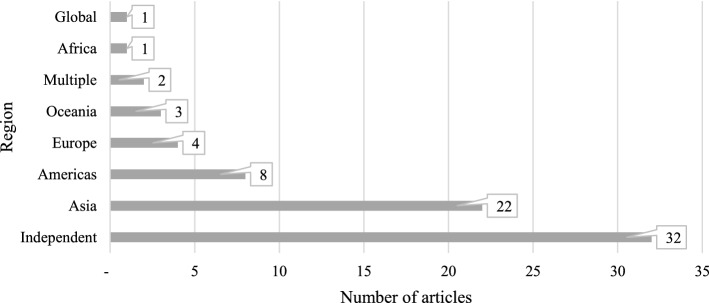
Region of disaster. *Source*: compilation by author

Disasters strike in any region, and no place is immune, especially when it comes to natural disasters. However, some regions are severely impacted by both economic damage and human casualties. The Asian region continues to be disaster-prone, and it is one of the world's most severely affected regions (Swiss Re, 2021), therefore it is expected that scholars will favour examining the events in this region, as shown in Fig. 7. The Americas are the second most studied region, however, academics prefer the United States over the South American region, with seven out of eight articles focusing on the United States, with a focus on hurricanes. One of the reasons for the high emphasis on the North American region is economic loss. Though human loss in North America is one of the lowest in the world, economic loss is the highest, accounting for nearly 52% of the overall world losses (Swiss Re, 2021). Other regions, Europe, Oceania, and Africa, have got much less attention. Africa has the second-highest number of disaster-related human mortality, behind Asia (Swiss Re, 2021), yet it is the least concentrated in this domain, where disaster and humanitarian assistance can be extremely crucial. Furthermore, while a couple of papers focus on multiple locations (Chaudhuri & Bose, 2020; Mulder et al., 2016), the vast majority are from the independent category, including some empirical and all conceptual articles where the investigation is not location-driven. Scholars studied disaster areas using a variety of technological platforms, including data processing and analytical tools Apache Spark (Avvenuti et al., 2018; Ragini et al., 2018), ScatterBlogs (Thom et al., 2016), and Weka (Kankanamge et al., 2020) along with programming languages such as R (Malawani et al., 2020; Sangameswar et al., 2017) and Python (Shan et al., 2019; Warnier et al., 2020).

**Fig. 8:**
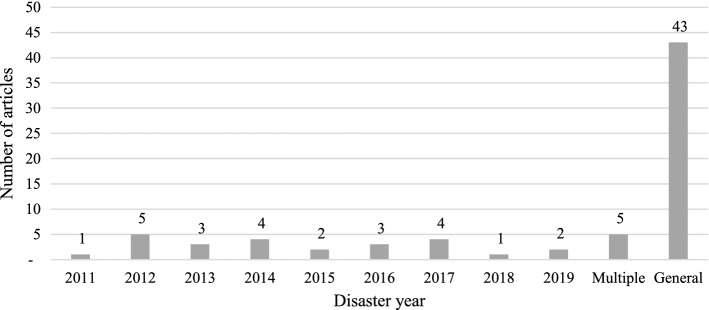
Year of disaster based on real cases in articles. *Source*: compilation by author

Figure 8 categorises disasters according to the year in which they occurred. The scholars picked disasters which happened between 2011 and 2019, with an average time gap between disaster incidents and research publication is three and half years. The year 2012 was one of the most expensive hurricane seasons in Atlantic history, and the fact that the most academic research selected disasters from the same year (as seen in the figure below) was due to an increase in scholarly interest in hurricane Sandy in the United States. Also, disaster that spanned across two different years were reported by more papers. As limited research is conducted by considering actual disasters as cases, the general category ends up with high number of publications that do not focus on real-world disasters. The remaining number of articles, which studied actual disasters, were broken down by regions in the “Appendix 2”, to discover which disasters were the most prominent in each region. Except for biological disasters, each group has at least one study on real disasters. Research on climatological disasters was only conducted in Europe and Oceania, whereas hydrological disasters are topped in the Asian region.

### Sources of big data

**Fig. 9:**
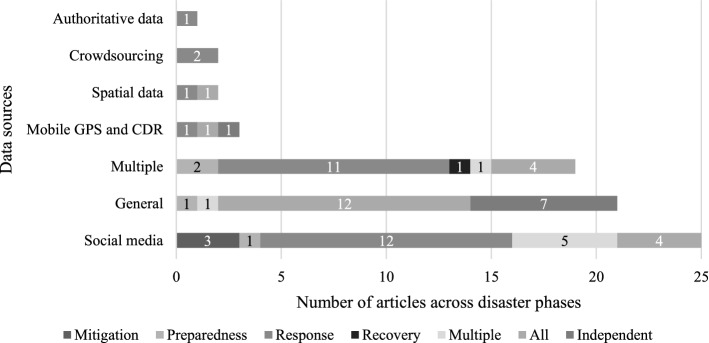
Big data sources across disaster phases in articles. *Source*: compilation by author

This review further examined the literature on the basis of data utilised for research, and Fig. 9 displays all of the big data sources investigated. Spatial data including satellite, aerial, and map data mainly serve as a visual aid for humanitarian and disaster responders, and researchers rely on these data sources to obtain greater accuracy (Lin et al., 2020; Nagendra et al., 2020). Ofli et al. (2016) chose aerial imaging over satellite imaging in their research because the processing time is shorter with aerial imagery. Aerial imagery is helpful for measuring small-scale disasters because it lowers cost, gives data in a timely manner, and avoids capturing difficulties because it is taken below the clouds (Meier, 2015). However, this will depend on the time and scale of the crisis, as satellite data will be beneficial for gathering precise texture information over a much larger area and approximately measuring height-related data, which can help quantify the damage intensity on the ground (Yu et al., 2018). When Mulder et al. (2016) examined crowdsourcing data in their study, they pointed out that by the time the data reaches the decision-makers, the original 'crowd' (often affected people) may have been eliminated from the information flow. In addition to their critical viewpoint, Givoni (2016) advocated for a cautious approach by studying two crowdsourcing platforms, the micro mappers and missing maps. Despite considerable technological developments, mobile phone data sources remain important and scholars explored the use of passive (positioning) and active (SMS) data to cover information gaps such as impacted people's location and need (Cinnamon et al., 2016; Nasim & Ramaraju, 2019). The most significant disclosure is the use of SM data with a staggering number of publications, which is not confined to developed countries, but is applied across every region, spanning all disaster phases, and being employed in majority of the disaster groups. Behl and Dutta (2019) work further confirms that scholars employed SM data extensively in their studies. A significant number of articles are general, with no emphasis on data sources, and a large proportion of them are conceptual studies.

Figure 9 also illustrates the comparison of the usage of various data sources across disaster phases, and this shows that academics preferred to use multiple data sources to better identify the needs of affected people at the disaster response stage than any other stage. To overcome the discrepancy when using multiple data sources, Griffith et al. (2019) stated that data must be cleaned to a significant extent, and cross-referencing between the sources must be performed. People responding to disasters often need to consider the impact of disasters on infrastructure, situation reports that update the ground reality, and information that describes risk levels, necessitating the use of several data sources (Warnier et al., 2020). In addition, scholars who studied the disaster response phase used all of the data sources shown in Fig. 9. The dominance of SM is not just in the response phase, but also when scholars studied multiple phases where it is the major source used for research. SM is not the preferred data source for examining disaster preparedness and recovery. People's engagement in SM typically grows from preparation to response and declines from response to recovery, according to Yan and Pedraza-Martinez (2019), while on some occasions scholars have turned to SM platforms during the mitigation phase.

Scholars preferred Twitter (20 articles) and Weibo (4 articles) within the SM data source to explore solutions to HDO-related challenges. Twitter's apparent dominance stems mostly from the fact that it includes huge volumes of publicly accessible data that is easy to comprehend, and most significantly, it offers timely data (Thom et al., 2016). Nevertheless, over-reliance on Twitter may pose bias-related concerns due to the extensive use of a single SM (Avvenuti et al., 2018), and data noise will be higher (Sherchan et al., 2017). Weibo, another SM network, has been featured in a few articles, but these studies have only been conducted in Asia. Furthermore, in one instance, academics used multiple SM data sources for their research, with Twitter being one among them. The utilisation of multiple SM networks as a data source, according to the researchers, provides a comprehensive view of the disaster's unfolding (Chaudhuri & Bose, 2020; Sherchan et al., 2017). Organisations may choose data sources in operations for a variety of reasons, including the best match for their circumstance in a disaster, availability or even financial capability to acquire data. While all three reasons appear rational, organisations should strive to select the first one, which is based on the best fit for the type of disaster, stage of disaster, and location of the disaster. Data cleansing and processing are key components of data analysis, accounting for 80% of overall data analysis (Griffith et al., 2019), therefore selecting an adequate data source is critical for operational efficiency.

### Theoretical underpinnings

The employment of theories is necessitated not just because of the intrinsic complexity of the HDO field, but also because of the context in which they occur (Galindo & Batta, 2013; Oloruntoba et al., 2019). The largest portion of the research in the examined articles did not apply any theories, and where they were used, there was no clear preference for one theory over another, not to mention that no single theory appeared more than once. The smaller percentage of theories in research publications might be attributed to the importance of applied research in the HDO field, where practitioners place a high value on practical relevance (Oloruntoba et al., 2019). The limited utilisation of theory has been mentioned in Akter and Wamba’s (2019) study, however, there appears to be a modest shift and improvement in theory usage over the last couple of years. Despite recent advances, the theory development in HDO is still in infancy, and there is a need and opportunity for researchers to integrate, expand, or even contradict theories to progress knowledge and overcome gaps (Oloruntoba et al., 2019).

Amaye et al. (2016) integrated organisational mindfulness processes with information system design theory to develop a mindfulness-based information systems assessment framework for making better decisions in emergency management circumstances. In an attempt to explain resilience, Papadopoulos et al. (2017) employed the TOSE resilience theoretical framework to investigate the use of big data in humanitarian supply chain networks for sustainability. From empirically testing their theory, authors demonstrated that the exchange of quality information in relief operations, public–private partnerships, and swift trust work as enablers of resilience in the humanitarian supply chain. In their research, Dubey et al. (2018) used a contingent resource-based view, in which the authors regarded big data predictive analytics as a capability for organisations that might be beneficial in visibility creation and coordination building, and swift trust could affect this relationship. Prasad et al. (2018) deployed resource dependence theory to investigate the interaction between non-governmental organisations (NGOs) and supply-chain partners and how this relationship affects the power dynamic in big data generation. According to the authors, partners in the supply chain have the ability to compel NGOs to employ BDA in their actions, which was empirically tested in three NGOs. Li et al. (2018), on the other hand, investigated population behaviour during disaster using a sociological theory called social exchange theory. The authors focused their investigation on people who were not impacted by the earthquake and if their actions on social platforms varied from those who were affected. Further, the organisational information processing theory was formulated into the humanitarian setting by Dubey et al. (2019) as an outcome. The authors empirically demonstrated that BDA capability has a favourable effect on both collaborative performance and swift trust.

Jeble et al. (2019) developed a conceptual model by interlinking two theories, resource-based view and social capital. In their work, authors developed a model based on big data and predictive analytics as a capability with tangible, intangible, and human resources, as well as social aspects such as trust, participation, social norms, and network to help improve performance in humanitarian supply chains. The road and distribution network will not be the same once the disaster strikes, because pre-disaster transportation models do not consider disaster-related disruptions. The use of social support theory in Yan and Pedraza-Martinez (2019) was to explore what elements inspire SM users to respond to the relief organisations’ posts during disasters and what form of social support the user interactions with organisations are connected with. The study by Warnier et al. (2020) utilises graph theory to investigate how to reach disaster-affected populations through these networks. Their research examined the transportation network using a variety of metrics, including centrality measurements, dynamic network properties, and intrinsic network properties. The work of Susha (2020) built a critical success factor (CSF) theoretical framework, found several elements for establishing data collaboratives, and streamlined them to the most relevant factors.

### Research methodologies

**Fig. 10:**
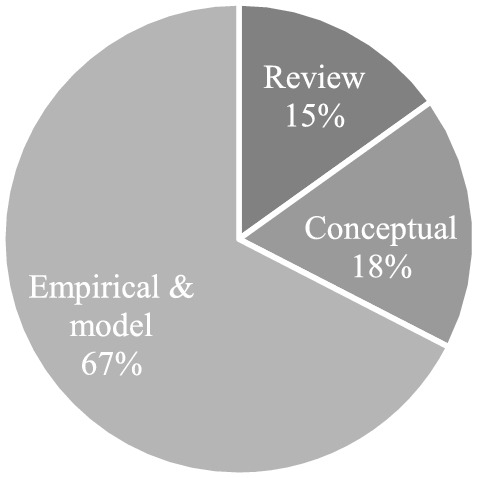
Research methods in articles. *Source*: compilation by author

We also looked into the research methodologies used in the articles to understand where the research is heading and what methods scholars prefer to study the field. Figure 10 depicts the distribution of articles into reviews, conceptual, and empirical and model papers. Though this is not a mature field, the amount of empirical and model research is predominant so far. In this, qualitative research (interviews, case studies, ethnography) is commonly used to study the activities engaged in the disaster response stage, but with a substantially lower ratio of theories used. On the other hand, quantitative research utilising surveys is minimal but studied events across various disaster phases, and the theory usage ratio is significantly better than qualitative studies. In addition, a few researchers employed mixed-method techniques with a focus on supply chain and situational awareness across disaster phases.

Scholars who employed models conducted extensive research on the Asian region, with half of them focusing on real-world disasters. Scholars developed models utilising MLP neural network, Apache Spark, and Python to better understand the needs of those affected. And relying exclusively on traditional approaches is no longer a viable option, thus scholars have either shifted to completely new data types or combined traditional data with new data types. In the segment of conceptual papers, scholars concentrated more on advocacy style in discussing the necessity of BDA or how to approach it, and a few papers highlighted difficulties of technology utilisation in the HDO. However, theory development or framework-related work is less visible in this part. “Appendix 4” shows the conceptual papers as well as the empirical & model studies. The review portion is discussed in length earlier in the 'review of reviews' section.

## Discussion

HDO management has progressed over the years in terms of establishing international mechanisms (UNDRR, 2015), assessing the needs for relief supplies (Apte et al., 2016), and even improving community-based disaster management (Zhang et al., 2013). Unfortunately, these developments have not been able to control the increasing number of fatalities, impacted populations, or economic losses, which have risen substantially from 1980–1999 to 2000–2019. Existing research that incorporates BDA into HDO focuses on disaster groups that are significant due to their frequency of occurrence, such as geophysical, hydrological, and meteorological. Concentrating entirely on frequent disasters does not help the field progress. Climatological and biological disasters should not be overlooked simply because they are less common than other disasters, and this does not imply that their impacts would be minimal. For instance, COVID-19, which began as an infectious disease has now evolved into an ongoing pandemic and a significant humanitarian crisis. If the early stages of an event are neglected, a hazard can turn into a disaster and a humanitarian crisis. Mami Mizutori, the UN Special Representative for Disaster Risk Reduction, has stated that “it is time to recognise that there is no such thing as a natural disaster” (UNDRR, 2021) highlighting that we bear a great deal of responsibility for resolving this, and our actions have to be decisive. Increasing research in less-focused disaster categories alone will not suffice. The disaster response phase is heavily concentrated in the context of BDA and HDO that the combined research of the other three phases mitigation, preparedness, and recovery makes up less than a third of the response phase. Mitigation and preparedness strategies have been widely debated as ways to lessen the negative effects of humanitarian crises and disasters (Asghar et al., 2006; Oteng-Ababio, 2013). This has been proven to be effective in terms of time and cost savings in two of the UN organisations' preparedness investments in three countries, according to pilot research conducted by Boston Consulting Group (2015). If that is so evident, why the research is not moving in this direction and investigate how BDA can bring additional value? Because, traditional HDOs are reactive, with relief agencies waiting for a disaster to unfold before initiating any humanitarian aid (Goldschmidt & Kumar, 2016). In 2020, UN OCHA launched a pilot programme called anticipatory humanitarian action in Bangladesh, using predictive analytics to intervene before the disaster (flood) occurred. As a result, more people were reached, aid became cheaper and faster, and the quality of assistance improved (UN OCHA, 2021). To see this initiative through in a central humanitarian agency, a lot of firsts had to happen, and such pilot projects at a larger scale won't be able to drive smaller organisations in the third sector. This could change if more research on pre-disaster phases involving local aid organisations is conducted.

Each HDO is distinct in its own right, just as each humanitarian crisis or disaster is unique in its own way. Every nation may not have the same emergency response systems, and the impacts will vary. Similarly, the research performed on each region differs in the field, with a substantial level of research in one region and a limited level of research in the other. However, the fact that Africa and South America were not represented in the 30 papers on actual disaster cases in review is cause for concern. Disasters and humanitarian crises pose a high to very high risk in Africa and a medium to high risk in South America (Thow et al., 2020). More focus in these regions, especially on Africa, would be particularly valuable because much of humanitarian work and the funding is directed here and the effective use of these funds is essential. The data availability and variations of multiple data sources can be a challenge in considering Africa and South America for research. Nonetheless, Humanitarian Data Exchange (HDX) currently includes data grids for 27 locations, 19 of which are in these two areas (Centre for Humanitarian Data, 2021). SM platforms remain popular data sources in examining the real case disasters and humanitarian crises (21 out of 30 articles), which fits well as long as privacy, ethical, and validation concerns are addressed. Though SM cannot be a one-size-fits-all solution for every data-related problem in HDO, The use of an additional data source, such as authoritative data, would complement SM data and could address validation concerns (Wang & Ye, 2018). A dialogue should be initiated to determine which big data sources are more suited to each disaster group and why, and how this can improve HDO efficiency. Based on existing research, a framework has been constructed in the “Appendix 3” that demonstrates what led scholars to select the specific big data source across several disaster groups. This was not possible in a few disaster groups due to a lack of empirical investigation, and for that reason, authors' views are included, which are indicated in bold in the same table. The framework was created by combining existing taxonomies on big data sources from UN Global Pulse (2012) and Qadir et al. (2016).

The scientific interest in the topic has increased greatly, and the year 2015 would be considered as an inflection point, with research moving at a breakneck pace since then. As a result, we opted to take stock of advancements in the field, as the years 2019 and 2020 had witnessed a tremendous amount of work. Though this is a multidisciplinary topic, and the work integration of multiple subject areas will only assist grow the field further so that practitioners could make better use of it, researchers from the management subject area can increase their attention towards this topic to holistically enrich it.

### Implications for practice

Some of the scholars highlighted how their study findings can be put into practice. Prasad et al. (2018) argued that third sector organisations must identify the important data attributes, as well as the change in expected results such as lead times and cost due to these data attributes, prior to the intervention. Yan and Pedraza-Martinez (2019) discussed how the usage of SM as a data source might be enhanced, and suggested that relief organisations use SM platforms for actionable information reaching volunteers, and donors. Furthermore, the use of SM as a big data source by public authorities should not be reactive, which necessitates a cultural shift in these organisations, and Zamarreño-Aramendia et al. (2020) made multiple recommendations on how SM can be used by the authorities. Fan et al. (2020) on the other hand, emphasised that response managers should consider the size of the population while employing SM to provide relief supplies to address spatial inequality. The work of Kontokosta and Malik (2018) on how the use of multiple big data sources can be helpful to reach the most affected people with a minimum capacity of resilience is noteworthy, and their REDI index is aimed at community organisations.

### Future research directions

Table 5 outlines possible directions for future studies from the standpoint of big data, through which a single or various big data sources can be employed to perform the research.

**Table 5:** Future directions to research in the field of BDA and HDO. *Source*: compilation by author

Big data source	Research directions (RD)	Research questions
SM	Research and practice reports validation-related concerns on the substantial usage of SM in HDO, which could result in operational inefficiencies. Though comprehensive population representation of disaster impact as a whole is always a challenge when using data, it is substantially higher with SM data. However, due to the data characteristics of SM, it is unlikely to completely avoid using it in disaster operations. Therefore, future research should investigate how validation-related concerns of SM can be addressed by HDO	Can a combination of additional big data sources be used to supplement SM to overcome validation related concerns?
		Are there characteristics of SM data that can assist in distinguishing between valid and in-valid data, both extracted from SM platforms? If yes, what approach can be adopted to identify or detect those characteristics?
		How can the geosocial challenge associated with population representative sample in SM, particularly with Twitter, be overcome?
	One of the repeatedly cited motivation for using SM as a data source in HDO is its ease of availability in comparison to other data sources. However, research has to investigate further to identify other reasons for frequent selection of SM data and compare it with reasons for selecting or not selecting other data sources	What are the motivations (in addition to easy availability) for adoption of SM as a data source in HDO?
		What data sources can humanitarian organisations and disaster relief agencies utilise outside SM and other than Twitter within SM?
		What do humanitarian organisations stand to gain and lose by utilising outside SM data sources and alternative sources other than Twitter within SM, especially in relief operations?
	Understanding public emotions during disasters and providing psychological relief is equally important as providing physical relief supplies and rescuing impacted individuals. SM has more information with the required data characteristics than any other big data source, future research can analyse them to understand the role they can play in delivering psychological relief and its impact on disaster response	What role can SM data play in providing psychological relief during the pre-disaster phases (when there is less SM activity) and how?
		What contributions could psychological relief data gathered from SM make to disaster response?
Multiple sources	The nature of traditional HDO is reactive because donors want to see how severe a humanitarian crisis or disaster is before allocating funds. Future research has to investigate the use of predictive analytics in pre-disaster stages involving local relief organisations. This study can contribute to further discussions about the use of BDA in the early stages of a disaster and whether it provides any benefits to the stakeholders involved	What factors should be taken into account to build the predictive model using multiple big data sources (authoritative data and data exhaust) even before a disaster occurs? To what extent may the application of BDA in the pre-disaster stages influence the effectiveness of relief operations?
	A considerable portion of academic research has investigated the HDO field by integrating multiple big data sources. In some circumstances, it was to supplement other data sources, but in others, it was absolutely vital. Relying on a single data source may become inadequate in the future. Research has to be undertaken to understand which combinations of big data sources are suitable for which disaster group	What challenges emerge when selecting specific big data sources for specific disaster categories? Is the selection of specific big data sources always situational or certain combinations can be discovered based on the characteristics of disasters? How to shortlist the best fitting data sources for different disaster categories?
All sources available	Each big data source may or may not possess all of the following characteristics: volume, variety, veracity, and velocity. Moreover, having these four characteristics in data may not be essential for some disasters or stages of disasters. The research can be carried out to identify the data characteristic requirements for distinct disasters and disaster stages, as well as to construct a framework to serve as a guide	What characteristics should big data sources possess across different stages of HDO? How do humanitarian organisations choose between one big data source over the other? How do organisations fill the voids caused by the absence of data characteristics in the available big data sources at a particular stage? How is the use of BDA distinguished between slow-onset and sudden-onset disasters?
	When evaluating the BDA in HDO, the disaster response stage stood out because it was the focus of the majority of the studies. Currently, the majority of disaster relief activities take place during the response stage, which is attributed to high logistical waste. A study is needed to further extend the claims of improved disaster response with the application of BDA, specifying what improvements and how much can be accomplished	What is the impact of BDA adoption on relief demand estimations? What tangible improvements can be expected in disaster response relief operations as a result of employing BDA? How can these tangible gains be measured in the context of disaster response?
	We need to understand if the use of BDA in local and national NGOs humanitarian operations in the global south assist them to be more cost-effective, less reliant on the global north, and also keep up with the north counterparts in delivering the aid. Research should examine the learnings from third-sector organisations that have already implemented big data in their HDO	What are the enablers of BDA adoption in local and national NGO’s? What are the similarities and differences in the adoption of BDA in local and national NGOs when compared to that of international NGOs?
	There seems to be little evidence or even conversation emerging from human-induced disasters, where there is more scope for anticipatory humanitarian action. It is essential to scientifically validate whether the BDA assists decision-makers in making rational decisions in the event of man-made disasters	What role do NGOs play in anticipating civil conflicts, and how might the data sources they possess enable them to engage in conflict prevention and resolution activities? In the event of a man-made crisis, what actionable information can be gained from situational awareness derived using BDA? What contributions can the BDA make to keep online extremism from escalating into offline violence?

In recent times, the most devastating disaster categories have been biological and climatological, while being mostly overlooked by academics, with just a fleeting reference in the current literature. The biological disaster group might receive a lot of attention from researchers in the coming years as a result of the COVID-19. Is it necessary to wait for a significant climate crisis to unfold before expanding this disaster group's research capabilities? Scholars need to bring attention to these understudied disaster categories in the natural disaster generic group, as well as level the research in the human-induced disaster generic group to evaluate how BDA may or may not be effective in certain disaster groups. If the knowledge gap between these two disaster generic groups widens further, this might lead to inconsistent suppositions and rationales for BDA in the HDO spectrum. Griffith et al. (2019) consider humanitarian logistics to be an immature field from an analytical standpoint because the solutions developed from the research efforts may not be employed in actual disaster settings due to computational burdens. This is something the academic community should take into account, rather than only developing models, they should strive to provide techniques, tools, and prospective solutions that can be used in real HDO settings.

## Conclusion

At the start of this review, 168 million people required various forms of humanitarian relief, by the end of the study, that figure had increased to 235 million. There is no time to waste, and certainly no data to be lost. Organisations in this field, such as NGOs, disaster management agencies, and other humanitarian societies, need to focus on exploring the use of BDA with the same tenacity as profit-driven enterprises while keeping ethical issues in check. Fortunately, academic research in this field is growing at a rapid pace, with the years 2019 and 2020 accounting for more than half of all research. Although significant progress has been made in the management subject domain, the total contribution has been minimal. Because it is a multidisciplinary field, various subject areas make important contributions. However, scholars from the management domain need to engage more in the advancement of the field. This study aimed to tackle three research questions and the topic in a systematic and more integrated manner. First, research on the application of BDA in HDO has substantially increased in recent years, demonstrating academics' interest and ability to investigate whether or not big data could improve the way humanitarian and disaster management operate. Second, the state of BDA application in the field remains lopsided among different disaster locations, disaster categories, and disaster stages, and research efforts were not utilised where they are more critical. Putting the emphasis on responding to disasters whilst overlooking the other three phases, mitigation, preparedness, and recovery will not lead to a comprehensive development of the field. Additionally, a heavy reliance on SM as a big data source has a factual, bias, and ethical concerns that need to be addressed. Third, a lack of theoretical frameworks is visible in the discipline; while this appears to be improving recently, the proportion of publications with a theoretical viewpoint in total papers published each year is not encouraging. Despite these significant findings, the review also has a few limitations, which the authors are aware of when undertaking the review.

### Limitations

There are three key limitations: one in database selection, one in exclusion criteria that was not part of the five-level search criteria, and one owing to the usage of SLR as a method. Though the selection of database is rational in this study, if the additional resources and time are available, web of science as an additional database could be incorporated for future studies. This addition may introduce a few more publications to the evaluation process and offer a much richer view of the subject. The second limitation is a Scopus-specific feature. To filter the results, the database provides two options: 'Exclude' and 'Limit to'. The subject area is one of the options to filter the results in Scopus, but it does not offer a unique split for papers by using the ‘Limit to’ condition. Because Scopus allocates each paper to several subject areas, it is not possible to get a unique number of articles listed in each subject area by using the 'Limit to' condition. The authors' reasoning for utilising this condition instead of 'Exclude' is that the 'Exclude' condition removes any publications with subject areas indicated in the 'Limit to' list (including subject areas in which the authors are particularly interested but will be omitted because each article contains tags of multiple subject areas). Furthermore, while the review was rigorous, it is possible that the author omitted a few studies because they did not fit the pre-defined inclusion/exclusion criteria.

## Data Availability

Not applicable.

## Appendix

### Appendix 3: The selection and rationale behind big data sources in each disaster group

**Table:**

	Authoritative data	Crowdsourcing data	Data exhaust	Online data	Sensor data
**Biological**	**• As a result of the ongoing biological disaster, the use of this data source is likely to increase in the future, and it can be supplemented with other data sources.**	**• This will help identify needs and resources, and in strengthening community resilience, which is currently being implemented through platforms like Ushahidi.**	• Helps in understanding the characteristics, and dynamics of the affected population. • High level of spatial and temporal resolution. • Population movements/mobility information. • Situational information. • Can produce risk maps.	**• This will be crucial in analysing public sentiment and behaviour. Enables humanitarian responders in tailoring their approach to crisis preparation and response.**	**• Sensor data generation will increase in the coming years, so the potential in biological disasters needs to be explored beyond tracking the population activities during a disaster.**
**Climatological**	• Damage predictions. • To monitor environmental information such as solar radiation, temperature, wind velocity, etc.	• Input from citizens reporting a fire occurrence.	**• This is largely owned by private entities; how entry barriers can be circumvented; and how passive data such as transactional data might be used in drought and wildfire disasters.**	• Enhance situational awareness. • Visual contextual information. • Information channel to citizens. • Accessible to people.	• Identifying vegetation. • Information on the area's slope and elevation. • Fire detection in the forest.
**Geophysical**	• Although this data (governmentproduced land, population, and shelter data) is not often used on its own, it is utilised in conjunction with other data sources.	• Developing situational awareness. • Capable of bridging information gaps. •Logistics network data. • Participation barriers are kept to a minimum.	• Infrastructure quality post-disaster. • Situational reports. • Damage assessments. • Population data.	• Information exchange behaviour. • Situational awareness. • Public emotions. • Community resilience. • Supply chain resilience. • Real-time data.	• Population distribution (spatiotemporal).
**Hydrological**	• Precipitation levels. • Rainfall forecasting. • Identifying the worst-affected areas. • Historical information on the impacted population, and relief supplies.	• The length of the rainfall. • Drainage capacity issue. • Intensity of rainfall.	• Location of affected individuals. • Safety checks can be monitored.	• Sentiment analysis. • Disaster severity. • Public safety. • Location-routing problem. • Situational awareness.	• Filling information gaps. • Estimating relief supplies. • Identifying logistics routes. • Identifying severity of disaster by area. • Co-ordination.
**Meteorological**	• To measure neighbourhood boundaries. • To obtain geographic delineation. • Damage and loss statistics.	**• The ground-level report is critical in sudden-onset disasters to better plan for and respond to crises, which is not addressed in previous research.**	• To identify the neighbourhood-level resilience capacity.	• Societal impacts. • People needs: change in priorities. • Sociodemographic factors. • Public behaviour.	• Geo-tagged human activities.
**Human-Induced**	• Personnel movements and locations. • Information flows related to missions.	• Actionable information.	**• Mobile CDRs, which are not currently being used in this context, will be able to generate near realtime data on the affected population. Other passive data adds to a better understanding of population behaviour at various stages of a crisis.**	• Situation awareness. • Identifying the location of the event.	• Improvement in decision support.

### Appendix 4: Methodological distribution of existing research

**Table:**

Conceptual	Empirical & model
Burns (2015), Sandvik et al., (2014, 2017), Read et al. (2016), Swaminathan (2018), Givoni (2016), Amaye et al. (2016), Burger et al. (2019), Fast (2017), Talley (2020), Gregg Greenough and Nelson (2019), Jeble et al. (2019), Tullis and Kar (2020), Gerdes (2020), Iglesias et al. (2020)	Papadopoulos et al. (2017), Rogstadius et al. (2013), Chae et al. (2014), Ragini et al. (2018), Ofli et al. (2016), Dubey et al., (2018, 2019), Landwehr et al. (2016), Mulder et al. (2016), Prasad et al. (2018), Cinnamon et al. (2016), Li et al. (2018), Kontokosta and Malik (2018), Avvenuti et al., (2017, 2018), Tomaszewski and MacEachren (2012), Kibanov et al. (2017), Zhang et al., (2016, ), Amato et al. (2019), Taylor (2016), Thom et al. (2016), Graham et al. (2015), van den Homberg et al. (2018), Shan et al. (2019), Sangameswar et al. (2017), Griffith et al. (2019), Yan and Pedraza-Martinez (2019), Kankanamge et al. (2020), Bahir and Peled (2016), Liu et al. (2020), Yang et al. (2019), Sherchan et al. (2017), Khoury (2019), Wang et al., (2019a, 2019b), Madianou (2019), Chaudhuri and Bose (2020), Susha (2020), Warnier et al. (2020), Nasim and Ramaraju (2019), Puttinaovarat and Horkaew (2019), Fathi et al. (2020), Abburu (2017), Nagendra et al. (2020), Malawani et al. (2020), Wu et al. (2020), Fan et al. (2020), Lin et al. (2020), Zamarreño-Aramendia et al. (2020), Romascanu et al. (2020), Qayum et al. (2020), Park et al. (2020), Zhang et al., (2020a, 2020b), Jin and Spence (2020), Akhtar et al. (2020), Bell et al. (2021), Bag et al. (2021)
